# Hemodilution causes glycocalyx shedding without affecting vascular endothelial barrier permeability in rats

**Published:** 2020-05-12

**Authors:** Bülent Ergin, Philippe Guerci, Zühre Uz, Martin Westphal, Yasin Ince, Matthias Hilty, Can Ince

**Affiliations:** ^1^Department of Adult Intensive Care, Erasmus MC, University Medical Center Rotterdam, Rotterdam, The Netherlands; ^2^Department of Translational Physiology, Academic Medical Center, University of Amsterdam, Amsterdam, The Netherlands; ^3^INSERM U1116, University of Lorraine, Vandoeuvre-Les-Nancy, France; ^4^Department of Anesthesiology and Critical Care Medicine, University Hospital of Nancy, France; ^5^Fresenius Kabi Deutschland GmbH, Bad Homburg, Germany

**Keywords:** acute normovolemic hemodilution,, fluids, glycocalyx, hydroxyethyl starch, microcirculation, vascular barrier permeability

## Abstract

**Background:**

The consequences of acute normovolemic hemodilution (ANH) following different types of fluids on the different components of the glycocalyx and on vascular barrier permeability (VBP) remain unknown.

**Aim:**

The aim of the study was to investigate whether the microcirculatory disruption and glycocalyx shedding induced by ANH alters VBP and whether this is affected by the composition and volume of the resuscitation fluid.

**Materials and Methods:**

Anesthetized Wistar albino rats (n=24) underwent stepwise ANH at hematocrit levels of 35%, 25%, 20%, and 15% induced by the exchange of blood with 6% balanced hydroxyethyl starch (1:1), balanced crystalloid (1:3), and normal saline (NS) (1:3). Glycocalyx-shed products were measured at each level of hemodilution. VBP was reflected in the decay of fluorescence dyes of different molecular size and their plasma retention ratios. Edema was assessed by measuring organ water content and muscle microcirculation by hand-held videomicroscopy.

**Results:**

NS caused increased degradation of heparan sulfate and hyaluronan compared with the control group (*P*=0.003, *P*=0.004, respectively). Neither VBP nor tissue edema was affected by the fluid used. The total and perfused vessel densities within the microcirculation of muscle tissue decreased at hematocrit 15% in the balanced crystalloid (*P*=0.02) and NS groups only (*P*<0.0001, *P*=0.0003, respectively) compared with baseline.

**Conclusions:**

Balanced colloid solution preserved the glycocalyx layer better than balanced and unbalanced crystalloid solutions while maintaining the microcirculatory function associated with an improved total intravascular volume. Among the fluids tested, NS caused the most microcirculatory alterations. While ANH caused the degradation of glycocalyx components regardless of fluid, it did not disrupt the vascular barrier as indicated by macromolecular leakage.

**Relevance for Patients:**

The results of this study provide insight into the choice of fluid for optimal perioperative fluid management and the consequences of fluid type on the vascular barrier, glycocalyx, and microcirculation.

## 1. Introduction

The optimal choice for perioperative fluid administration has been a source of debate for the past 40 years [1,2]. In the perioperative setting, acute normovolemic hemodilution (ANH) is required to compensate for blood loss or hypovolemia [3,4]. However, it is well-known that the composition and volume of the fluids being administered affect the endothelial cells, tissues, and organs potentially leading to coagulopathy, edema, hypoxia, endothelial damage, and acidosis.

The glycocalyx is an endothelial cell lining composed of highly negatively charged glycosaminoglycans that provide a protective layer and regulates leukocyte to endothelial cell adhesion and direct macromolecular trafficking. In a human clinical setting, ANH does not affect the glycocalyx constituents, in contrast to hypervolemia, due to the action of atrial natriuretic peptides [5]. The disruption of the glycocalyx is the primary cause of increased vascular barrier permeability (VBP), which leads to secondary tissue edema and other unfavorable outcomes [6]. However, considering that VBP tightly regulated, the importance of the contribution of the glycocalyx to VBP has been questioned recently in relation to the other constitutive elements [7].

Different types of fluid used for increasing blood volume or compensating for intraoperative blood loss (leading to ANH) impact the glycocalyx in different ways. Colloid solutions preserve glycocalyx components by maintaining the shear stress on the endothelium with its higher viscosity [8] and osmotic pressure. However, the degree to which glycocalyx shedding is affected by stepwise ANH with different types of fluid has not been investigated. In addition, the effect of ANH on VBP has not yet been described.

The aims of the present study are: (i) To investigate which of the different components of the glycocalyx are shed in response to the fluids commonly used perioperatively, and (ii) to test the impact of glycocalyx degradation on microcirculatory parameters and VBP in stepwise ANH.

## 2. Materials and Methods

### 2.1. Animals

This study was approved by the Animal Research Committee of the Amsterdam University Medical Center (DFL 190AA). All procedures were performed in accordance with the guidelines of the Institutional Animal Care and Use Committee and the Declaration of Helsinki. Data are reported in line with the ARRIVE guidelines for reporting *in vivo* experiments. Experiments were performed in the Department of Translational Physiology (Amsterdam University Medical Center, Amsterdam, the Netherlands) on male Wistar albino rats (Charles River Laboratories, the Netherlands), aged 10±3 weeks, with a mean±SD bodyweight of 309±17 g.

### 2.2. Surgical preparation

Anesthesia was induced with an intraperitoneal injection of 100 mg/kg ketamine (Nimatek; Eurovet, the Netherlands), 0.5 mg/kg dexmedetomidine (Dexdomitor; Pfizer, NY, USA), and 0.05 mg/kg atropine-sulfate (Centrafarm, the Netherlands) and sustained with continuous intravenous infusion of 50 mg/kg/h ketamine. Fluid levels were maintained with Ringer’s acetate solution (Baxter, Utrecht, the Netherlands) at a rate of 10 ml/kg/h. After the tracheostomy, the animals were connected to a ventilator (Babylog 8000, Dräger, the Netherlands) set to tidal volumes of 6 ml/kg and a positive end-expiratory pressure of 3 cmH_2_O and FiO_2_ of 0.4. A heating pad was used to maintain the animals’ body temperatures at 37±0.5°C. End-tidal CO_2_ was kept between 30 and 35 mmHg (CapnoMac, Datex-Ohmeda, USA) by adjusting ventilator settings. A detailed description of the surgical procedure is available in the literature [7].

### 2.3. Experimental protocol

Animals (n=6/group) were randomized according to a unique code generated by an internet website (http://www.sealedenvelope.com). On the 1^st^ day of the experiment, a technician prepared the fluid used for the hemodilution protocol according to the generated code. After a 30-min stabilization period, baseline hemodynamic values, including mean arterial pressure (MAP), were recorded, and arterial blood gas levels were assessed (ABLFlex 80, Radiometer, Denmark).

**Figure 1:**
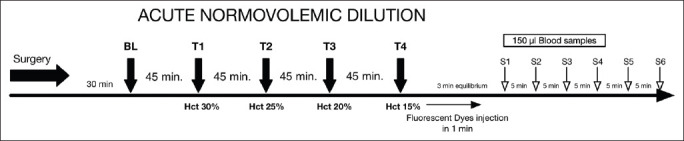
Time course of the acute normovolemic hemodilution.

ANH was induced in three groups by the stepwise exchange of blood (withdrawn at a rate of 0.5 ml/min) using a syringe pump (Harvard Apparatus, USA) and exchanged with one of the following plasma expanders according to the following ratios: 1:1 for 6% balanced hydroxyethyl starch (HES) (Volulyte 6% HES 130/0.4 in Ringer’s acetate), 1:3 for balanced crystalloid (Ringer’s acetate), and 1:3 for normal saline (NS) 0.9% until successive target levels of hematocrit were reached, approximately 45 min each. All solutions were purchased from Fresenius Kabi (Germany). A fourth group was instrumentally prepared but received no fluid and served as a time control group. Each stepwise hemodilution targeted four hematocrit levels: 35%, 25%, 20%, and 15%. The time course of the experiment is presented in Figure 1.

### 2.4. Biomarkers of glycocalyx shedding

Circulating plasma syndecan-1, heparan sulfate, and hyaluronan were measured as surrogates for glycocalyx. The stored plasma samples were thawed and analyzed using commercial enzyme-linked immunosorbent assay (ELISA) kits according to the manufacturer’s instructions: DuoSet Hyaluronan (DY3614, R&D Systems, Minneapolis, MN, USA), Syndecan-1/CD138(SCD1) ELISA Kit (Cusabio Biotech, Wuhan, China), and Heparan Sulfate Proteoglycan 2 (HSPG2) ELISA Kit (Cusabio Biotech) were used for this purpose.

### 2.5. Skeletal microcirculatory measurements

A hand-held vital microscope with incident dark-field (IDF) imaging, CytoCam (Braedius Scientific, Huizen, the Netherlands) was placed on the surface of the exposed biceps femoris to continuously monitor the microcirculation. One hundred frame clips (100 s) were recorded at every time point for each hematocrit level. All clips were randomly anonymized, and the microcirculation was analyzed blindly (group and time point) at baseline and each hemodilution time point using Automated Vascular Analysis 3.2 (Microvision Medical, Amsterdam, the Netherlands). Recorded parameters included total vessel density (TVD), perfused vessel density (PVD), the proportion of perfused vessels (PPV), and mean flow index (MFI), as previously described [9,10].

### 2.6. Assessment of VBP and tissue edema

#### 2.6.1. Fluorescent tracers

A fluorescence-based VBP analysis was performed as previously described [7]. Fluid maintenance was halted at the end of the experiment. Three fluorescent dyes conjugated with different sized molecules and dissolved in saline were thoroughly mixed and manually injected intravenously over 1 min: Texas-Red-40 kDa dextran (10 mg/ml, D1829, Thermo Fischer Scientific, Breda, the Netherlands), albumin-Alexa 680 (albumin 70 kDa) (5 mg/ml, A34787, Thermo Fischer Scientific, Breda, the Netherlands), and fluorescein isothiocyanate (FITC)-500 kDa dextran (10 mg/ml, MFCD00131092, Sigma-Aldrich, Zwijndrecht, the Netherlands) (100 μl each). The blood samples (200 μl) were withdrawn at 2, 5, 10, 15, 20, 25, and 30 min to measure the decay in plasma concentration of the dyes, as described in detail in the literature [7]. Plasma concentrations were determined for each dye using a 96-well plate fluorometer (ClarioStar, BMG LabTech, Ortenberg, Germany) in accordance with the excitation/emission wavelengths of each dye and a standard calibration curve. Measured wavelengths included 580±20 nm/625±20 nm for FITC, 480±15 nm/530±25 nm for Texas-Red, and 675±10 nm/740±40 nm for Alexa 680. The retention ratio (RR) of each dye was calculated as follows: RR=final concentration at 30 min/initial concentration at 2 min. For total intravascular volume (TIV) and plasma volume (PV) determinations, the concentration of albumin-Alexa 680 was determined 2 min after the bolus injection according to the following equation:


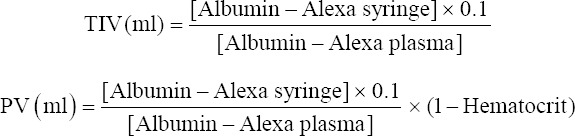


Hematocrit is expressed as percentage (e.g., 15%=0.15). “0.1” corresponds to the volume (in ml) of albumin Alexa 680 contained injected from the syringe.

#### 2.6.2. Tissue edema

At the end of the experiments, organs (heart, brain, kidney, lung, and liver) were harvested to determine their water content. The organs were placed in an oven at 100°C for 24 h. The wet-to-dry weight ratio was calculated as follows: Wet tissue weight/dry tissue weight.

### 2.7. Statistical analysis

nQuery Advisor was used to determine that, for a sample size of six per group, 80% power is required to detect a MAP difference of 46 mmHg between the control and hemodiluted groups with an average standard deviation (SD) of 25 using a two-group t-test with α=0.05 two-sided significance level.

Values are expressed as mean±SD when normally distributed (Kolmogorov–Smirnov test) or as median (interquartile range) when not. Ninety-five percent confidence intervals (95% CI) are reported when necessary. Repeated measures two-way analysis of variance (RM-ANOVA) (two factors: Time as the related within-animal factor and group as the between-animal factor) with Bonferroni’s *post hoc* multiple comparisons test were used to determine inter- and/or intra-group differences in hemodynamic, microcirculatory, biochemical, and glycocalyx degradation biomarkers. When a significant interaction was observed between time points and groups, the main effect of group (fluid type) compared with the control and balanced HES groups at each time point was reported. The simple main effect of time versus baseline within the same group was also reported. One-way ANOVA with Bonferroni’s correction was used for the RRs. Because of their non-Gaussian distribution, the Kruskal–Wallis test was used with Dunn’s post-test. The fluorescence values from each time point were fitted with an exponential one-phase decay curve using the least-squares method. Statistical analysis was performed using GraphPad Prism version 8.1.2 for Mac (GraphPad Software, La Jolla, CA, USA).

For comparison of microcirculatory parameters relative to baseline and hemodilution time points, two-way linear mixed-effects model analysis was used (R, using the R library *lme4*, version 1.1.13). Subgroups for which a statistically significant effect was detected were further analyzed using a one-way linear mixed-effects model. The overall significance level used for each hypothesis was 0.05. Adjusted *P*-values were reported throughout the manuscript for the *post hoc* tests. No a priori power analysis was performed.

## 3. Results

### 3.1. Systemic hemodynamics and blood variables

All rats (n=24) survived the stepwise ANH procedure. However, ANH was associated with a significant decrease in MAP in all groups compared with control, which peaked at hematocrit 15% (F [4, 20]=113.7, *P*<0.0001). The hemodynamic data are summarized in Table 1.

**Table 1:** Hemodynamics, blood hematocrit, hemoglobin, lactate, and arterial partial pressure of CO_2_ levels throughout experiment

		Baseline		Hct 30%		Hct 25%		Hct 20%		Hct 15%
MAP (mmHg)										
Control		92±14		80±4		80±11		80±10		84±10
Balanced HES		95±10		66±1*		60±1*		58±4*		56±5*
Balanced crystalloid		87±10		66±11*		54±4*		54±8*		47±7*
NaCl % 0.9		90±12		61±5*		56±5*		50±2*		42±5*
CVP (mmHg)										
Control		7±1		7±1		6±2		6.5±1.5		7±1
Balanced HES		6±1		6±1		7±1		6.9±1.3		7±1
Balanced crystalloid		6±1		7±1		7±2		7±2		7±2
NaCl % 0.9		5±1*		5±1*		6±1^#^		6±1^#^		6±1*^#^
Hemoglobin (g/dl)										
Control		14.2±0.5		13.5±1.1		12.9±1		13.6±1.1		12.2±1.3
Balanced HES		14.6±0.8		9.6±0.5*		7.9±0.7*		6.2±0.4*		4.6±0.4*
Balanced crystalloid		14.9±0.8		9.9±0.9*		7.8±0.6*		6.2±0.3*		4.7±0.4*
NaCl % 0.9		14.3±1.4		10.1±0.3*		8±0.4*		6.5±0.5*		4.8±0.2*
Hematocrit (%)										
Control		44±1		41±3		40±3		41±4		37±4
Balanced HES		45±2		30±2*		25±1*		20±1*		15±2*
Balanced crystalloid		46±3		31±2*		25±2*		20±1*		15±1*
NaCl % 0.9		45±4		31±1*		25±1*		21±1*		15±1*
pH										
Control		7.42±0.05		7.40±0.02		7.42±0.03		7.42±0.02		7.41±0.02
Balanced HES		7.45±0.03		7.44±0.03		7.44±0.04		7.40±0.03		7.39±0.03
Balanced crystalloid		7.44±0.03		7.41±0.02		7.40±0.02		7.40±0.02		7.36±0.02*
NaCl % 0.9		7.46±0.03		7.38±0.03		7.34±0.03*^#^		7.31±0.03*^#^		7.29±0.05*^#^
HCO^-^_3_ (mmol/L)										
Control		21.3±1.7		20.1±2		18.9±0.8		19.2±1.4		20.4±1.2
Balanced HES		20.9±1.2		23.1±1.2*		24±1*		23.5±1.5*		23±1.6*
Balanced crystalloid		20.8±2.2		22±2.1*		22.7±1.5*		21.9±1.7*		22.7±1.8*
NaCl % 0.9		20.4±2.4		19.8±1.5^+#^		19.1±1.5^+#^		18.4±1.5^+#^		16.8±1.5*^+#^
Lactate (mmol/L)										
Control		0.9±0.3		1.2±0.3		1.2±0.6		1.5±0.5		1.5±0.5
Balanced HES		0.9±0.2		1.1±0.3		1.3±0.5		2.1±0.8*		2.7±1*
Balanced crystalloid		1.3±0.3		1.4±0.6		1.9±0.7*		2.4±0.7*		2.4±0.7*
NaCl % 0.9		1.1±0.2		1.1±0.7		1.4±0.3*		1.6±0.4^#^		2.5±0.5*
PaCO_2_ (mmHg)										
Control		34±5		33±4		30±1		30±1		33±3
Balanced HES		31±3		35±4		36±4*		39±3*		39±2*
Balanced crystalloid		31±3		36±5		37±3*		36±3*		41±3*
NaCl % 0.9		30±3		35±3		36±4*		38±5*		36±3^#^

Hct: Hematocrit, MAP: Mean arterial pressure, HCO_3_ −: Bicarbonate, CVP: Central venous pressure, HES: Hydroxyethyl starch. Data are presented as mean±SD. *Adjusted *P*<0.05 versus control group, # adjusted *P* value versus balanced HES group

**Figure 2:**
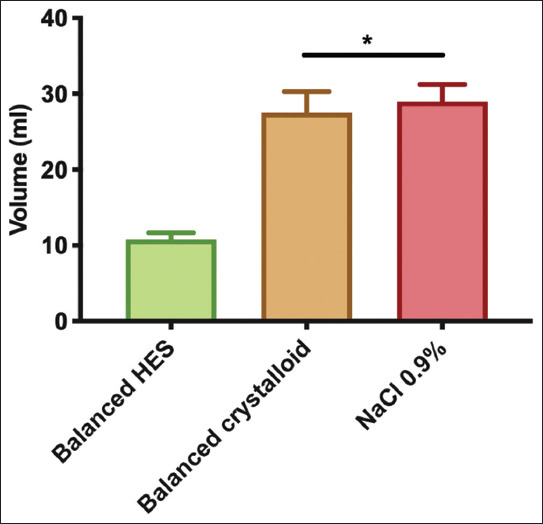
Total fluid volumes during acute normovolemic hemodilution. Ordinary One-way ANOVA test with Bonferroni’s correction to adjust for multiple comparisons. *adjusted *P*<0.0001 versus Control group (n=6/group).

Stepwise ANH with 0.9% saline induced progressive metabolic acidosis (F [3, 15]=16.89, *P*<0.0001) accompanied by changes in pH and bicarbonate levels relative to control (7.29±0.05 vs.7.41±0.02, mean diff. 0.12, 95% CI [0.09; 0.15], *P*<0.0001) (Table 1). At a hematocrit level of 15%, ANH was significantly associated with hyperlactatemia regardless of the fluid administered (2.7±1 mmol/l, mean diff. −1.27, 95% CI [−1.81; −0.72] for balanced colloid, 2.4±0.7 mmol/l, mean diff. −0.95, 95% CI [−1.49; −0.41] for balanced crystalloid, and 2.5±0.5 mmol/l, mean diff. −0.85, 95% CI [−1.39; −0.31] for NS, *P*<0.001) compared with control (1.5±0.5 mmol/l). The total volume of balanced HES required during the experiment to achieve each step of ANH was significantly lower (10.8±0.9 ml) than in the balanced crystalloid (27.5±2.8 ml) and NS (29±2.2 ml) groups (*P*=0.0001) (Figure 2).

**Figure 3:**
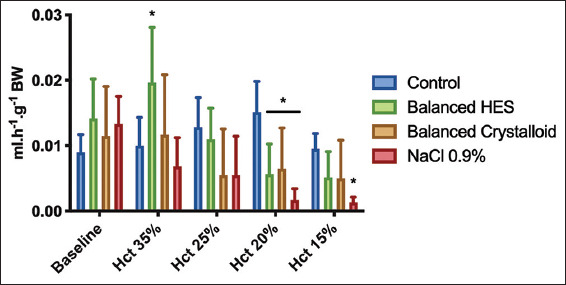
Urine output during acute normovolemic hemodilution. Repeated measures two-way ANOVA test used with Bonferroni’s correction to adjust for multiple comparisons. *adjusted *P*<0.01 versus control group at the same time point (n=6/group).

Urine output decreased with hematocrit level in all groups compared with baseline (F [4, 20]=15.35, *P*<0.0001) (Figure 3). However, at a hematocrit level of 15%, only ANH with 0.9% saline group exhibited significantly decreased urine output compared with the control group (0.001±0.001 vs. 0.01±0.002 ml/g BW^−1^, mean diff. 0.008, 95% CI [−0.001; 0.017], *P*=0.03).

### 3.2. Plasma and TIV

**Figure 4:**
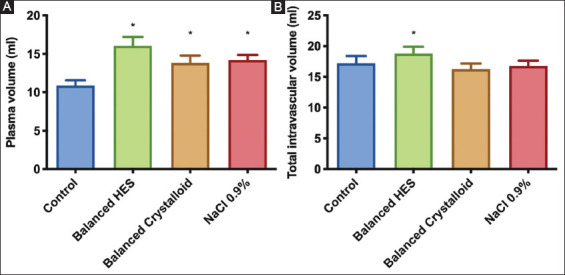
Plasma and total intravascular volumes at the end of experiments determined by measurement of albumin Alexa. Ordinary one-way ANOVA test with Bonferroni’s correction to adjust for multiple comparisons. *adjusted *P*<0.001 versus control group (n=6/group).

PV increased in all groups compared with control (F [3, 20]=35.68, *P*<0.0001) (Figure 4A). However, TIV (Figure 4B) only increased in the balanced HES group (18.8±1.1 ml) compared with control (17.2±1.2 ml) (mean diff. −1.6, 95% CI [−3.1; −0.07], *P*=0.039). Despite similar hematocrit levels in all ANH groups (Table 1), only hemodilution with balanced HES increased the intravascular volume.

### 3.3. Glycocalyx degradation biomarkers

**Figure 5:**
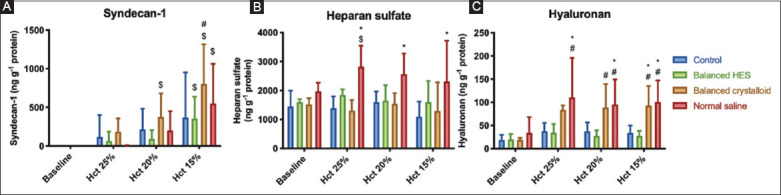
Plasma levels of syndecan-1 (panel A), heparan sulfate (panel B), and hyaluronan (panel C) during experiments. Increased glycocalyx shedding persists after fluid administration except for the hyaluronan component, which decreases when balanced HES is used. RM-ANOVA test used with Bonferroni’s correction to adjust for multiple comparisons. *adjusted *P*<0.01 versus control group at the same time point, # *P*<0.05 versus balanced HES group, $ *P*<0.05 versus baseline value within the same group (n=6/group).

Syndecan-1 was shed throughout the experiment in all groups including control. At the end of experiment (hematocrit 15%), syndecan-1 levels were significantly higher than baseline (F [3, 60]=23.88, *P*<0.0001) (Figure 5A). ANH with NS resulted in significant shedding of heparan sulfate (Figure 5B) and hyaluronan (Figure 5C) compared with control (mean diff. −1213, 95% CI [−2070; −356], *P*=0.003 and −66, 95% CI [−115; −18], *P*=0.004, respectively). Similarly, balanced crystalloid solution altered the hyaluronan (mean diff. −59, 95% CI [−107.6; −10.33], *P*=0.013) but not heparan sulfate levels. ANH using balanced HES only induced minimal changes to the levels of the components shed from the glycocalyx.

### 3.4. VBP

#### 3.4.1. Fluorescent tracers in the vascular compartment

**Figure 6:**
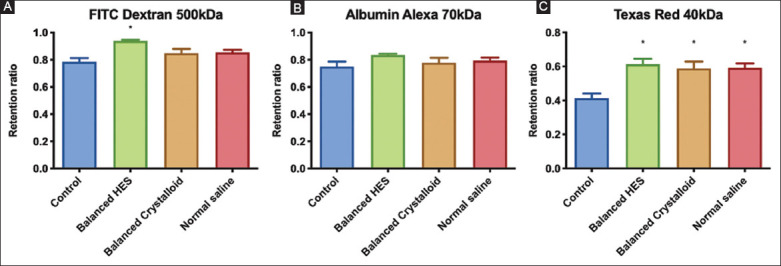
Retention ratios of fluorescent dyes at the end experiments. Ordinary one-way ANOVA test with Bonferroni’s correction to adjust for multiple comparisons. *adjusted *P*<0.05 versus control group (n=6/group).

The RRs of the fluorescent dyes injected into the vascular system after the last ANH time point are shown in Figure 6. Thirty minutes post-injection, the smallest fluorescent dye (Texas-Red 40 kDa) was significantly retained within the vasculature independent of the fluid administered (F [3, 19]=7.529, *P*=0.0016). The balanced HES group showed the highest Texas-Red 40 kDa RR compared with the control group (0.61±0.08 vs. 0.41±0.06 mean diff. −0.2, 95% CI [−0.32; −0.08], *P*=0.0012). These results indicate an absence of increased vascular leakage in ANH.

**Figure 7:**
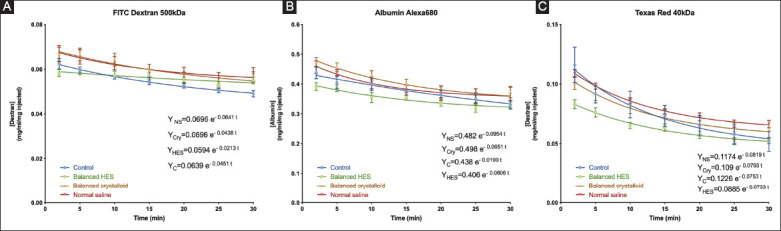
(A-C) Decay traces of each fluid group classified by fluorescent tracer.

Figure 7 shows the decays curves associated with each fluid group according to each fluorescent tracer. Compared with RRs, the powers of the exponential decay curves only tended to be different between fluids and control groups with regard to the Texas-Red 40 kDa dye but did not reach statistical significance.

#### 3.4.2. Tissue edema

**Figure 8:**
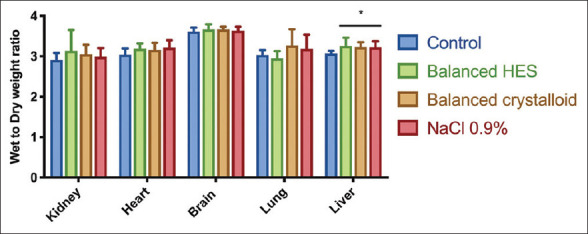
Edema in different organs assessed by the wet to dry weight ratio *adjusted *P*<0.05 versus control group (n=6/group).

The water content of each organ using the wet-to-dry weight ratio method is summarized in Figure 8. Except in the liver, no significant tissue edema was present post-ANH regardless of the type of fluid administered.

### 3.5. Microcirculation of the muscle during stepwise ANH

**Figure 9:**
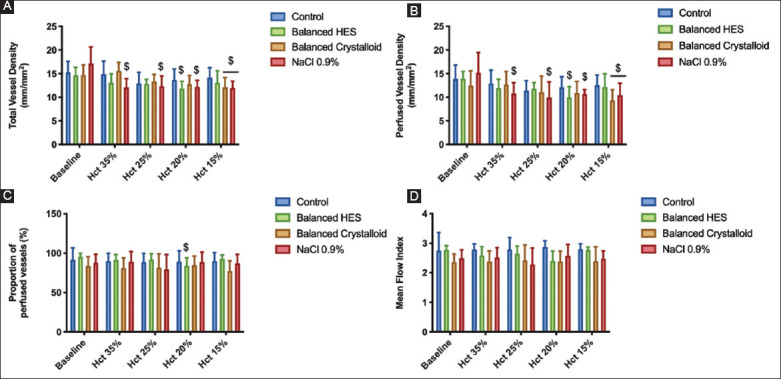
Microcirculatory muscle alterations at each step of acute normovolemic hemodilution. Total vessel density (Panel A), perfused vessel density (Panel B), proportion of perfused vessels (Panel C), and mean Flow Index (Panel D) are presented.

Figure 9 depicts the microcirculatory changes occurring during ANH according to the type of fluid used. Baseline values within each group, ANH altered both TVD and PVD. ANH with balanced colloid altered the microcirculatory parameters a lesser degree of especially with regards to PVD. The maximum decrease in TVD and PVD were observed at a hematocrit level of 15% in the crystalloid groups, with mean differences of 3.1, 95% CI (0.32; 5.9), *P*=0.02 and 4.7, 95% CI (1.9; 7.5), *P*=0.0003 for balanced crystalloid and NS groups, respectively, compared with baseline. No changes were detected in MFI or PPV compared with baseline.

Two-way linear mixed effects model analysis was used. $ *P*<0.05 compared to baseline within the same group (n=6/group).

## 4. Discussion

In this translational study, ANH with balanced HES prevented the shedding of different components of the glycocalyx. However, crystalloids, either balanced or not, significantly shed glycocalyx components, namely, syndecan-1 and hyaluronan, in comparison to balanced HES. Only ANH with NS resulted in the degradation of all three main constituents of the glycocalyx. However, despite glycocalyx degradation following maximum ANH, no increase in VBP was observed, regardless of fluid type and composition. While ANH at a hematocrit level of 15% induced microcirculatory disturbances, which was associated with impaired macrohemodynamics, these were partially prevented by the use of balanced HES solution compared with crystalloids.

These results are consistent with the previous studies showing that low plasma and blood viscosity may dampen the endothelial cell response to shear stress and subsequently alter the vascular tone [11,12]. Guerci *et al*. demonstrate that plasma and blood viscosity were similarly impacted following resuscitation after non-traumatic hemorrhagic shock with either low-(Ringer’s lactate) or high-viscosity (HES) plasma expanders [13]. The glycocalyx participates in endothelial shear stress-induced reactivity by sensing the flow and mediating mechanotransduction signals, mainly through syndecan-1 [12,14]. In the present study, syndecan-1 was the most shed component following the use of NS. Moreover, macrohemodynamics were severely impaired at the final stage of ANH with a MAP of 42±4 mmHg in the NS group. In the present study, extreme ANH-induced microcirculatory disturbances were associated with hypotension, endothelial glycocalyx degradation, and vascular hyporesponsiveness, as previously reported [11], but did not alter the VBP.

The absence of increased VBP following ANH suggests that a component of the vascular barrier, such as intercellular junctions, can withstand the transendothelial water flux despite partial glycocalyx degradation [15]. Thus, the typical transendothelial flux of proteins and water in and out of the interstitial space can be maintained during ANH. Moreover, in agreement with our findings, Rehm *et al*. found that, in addition to the glycocalyx, another component of the vascular endothelial barrier formed by endothelial cells (“double-barrier” concept) may play a role in fluid maintenance and colloid extravasation in an isolated heart model [16]. In the present study, we showed that small molecules (Texas-Red dextran-40 kDa) did not escape more rapidly from the vascular compartment than larger ones, as suggested by the similar exponential decay curves fitted to the plasma concentrations of the dyes, regardless of the fluid used for ANH. In addition, the RRs in all ANH groups were higher than the control group, which confirms the absence of vascular barrier disruption during ANH. Compared with the diluted group, the low RR of 40 kDa molecules in the control group indicated more leakage of small molecules from the vascular to the interstitial space under baseline conditions. The viscosity and shear stress created by the red blood cells under baseline conditions could contribute to the permeability of the vascular barrier to provide osmotic balance in intra- and extra-vascular spaces. However, blood viscosity and shear stress reduced by the removal of red blood cells may also decrease the permeability of small molecules through the vascular barrier to maintain intravascular volume. One would expect a decrease in RRs for small molecules due to the increased VBP of water and proteins. These results imply that, regardless of the type of fluid administered, water will relentlessly escape from the vasculature either by urine production or by transendothelial outflux. For acute kidney injury, the latter is more likely to occur. However, in the present study, no fluid accumulation with the wet-to-dry weight ratio technique was evidenced in various organs except in the liver despite having given 3 times higher volume of crystalloids than colloids to achieve the same PV between groups. Due to reduced urinary discharge, this extra volume of crystalloids, especially saline, might accumulate in the third space.

The shedding of the glycocalyx has been reported in human clinical settings and in preclinical models, as a response to ischemia/reperfusion injury [17], trauma [18,19], sepsis [20], and even during hyperglycemia [21] and after smoking [22]. In a clinical intraoperative setting, Chappell *et al*. compared the effects of volume loading (top-loading) and ANH on the presence of glycocalyx shed products in blood plasma using HES 130/0.4 during elective surgery in patients with healthy heart function [5]. In contrast to ANH, volume loading induced a significant release of atrial natriuretic peptide that was associated with increased plasma hyaluronan and syndecan-1 levels. These findings are partially consistent with our results. Although PVs were markedly increased secondary to ANH in all groups compared with the control group, the TIV only increased in the balanced HES group. Finally, balanced HES was less likely than crystalloids to induce the shedding of heparan sulfate, hyaluronan, and syndecan-1 despite the increase in PV, which renders it more suitable for maintaining the integrity of the glycocalyx during ANH.

At present, ANH remains well-recognized as a safe procedure for perioperative patient management [3,4]. Unintended ANH may also result from intraoperative fluid administration. Increasingly more patients develop anemia during the post-operative course, secondary to unintentional ANH, in response to restrictive red blood cell transfusion with no apparent negative outcome [23,24]. Even if ANH with NS or balanced crystalloids were to negatively impact the glycocalyx, the clinical consequences of this degradation remain undetermined.

### 4.1 Limitations

First, VBP was analyzes using only two assays. Previously, we demonstrated similar results in a non-traumatic hemorrhagic shock model in rodents using four different techniques [7]. Second, we did not investigate glycocalyx ultrastructure by electron microscopy or *in vivo* fluorescent microscopy. We relied on surrogates for glycocalyx shedding, which may represent a limitation. Third, we did not analyze the consequences of hemodilution on microcirculatory oxygen availability in the different organs to determine which fluid provides the best tissue oxygenation because a similar study has already been published [25]. Fourth, due to technical and ethical reasons, we did not analyze glycocalyx and endothelial barrier function after longer periods of hemodilution.

## 5. Conclusions

The use of a balanced colloid solution preserved the glycocalyx, maintained microcirculatory function, and improved TIV compared with both balanced and unbalanced crystalloids. NS had a more deleterious effect than the balanced crystalloid and colloid on macro- and micro-circulation during ANH. Despite the volumes required to achieve similar ANH in all groups being different, no evidence was found of increased VBP or organ edema following ANH. Consistent with our previous study in non-traumatic hemorrhagic shock, the results of this study suggest that glycocalyx shedding may induce the loss of vascular tone in response to shear stress without subsequent increment of VBP as a result of intercellular vascular barrier disruption.

### Funding

The study has been supported in part by Fresenius Kabi. This study has been also supported by funds from the Department of Translational Physiology, Academic Medical Centre, Amsterdam, The Netherlands. Philippe Guerci is supported by a grant from the Société Française d’Anesthésie et de Réanimation (SFAR).

### Conflicts of interest disclosure

Dr Can Ince runs an internet site microcirculationacademy.org which offers services (e.g., training, courses, and analysis) related to clinical microcirculation and, has received honoraria and independent research grants from Fresenius-Kabi, Baxter Health Care, and AM-Pharma; has developed SDF imaging; is listed as an inventor on related patents commercialized by MicroVision Medical under a license from the Academic Medical Centre; and has been a consultant for MicroVision Medical in the past but has not been involved with this company for more than 5 years. The company that developed the CytoCam-IDF imaging system, Braedius Medical, is owned by a relative of Dr Ince. Dr Ince has no financial relationship with Braedius Medical (i.e., never owned shares or received consultancy or speaker fees). Prof. Martin Westphal is chief medical officer of Fresenius kabi. The remaining authors declare no conflicts of interest.
